# Exploring the additive effect of Ampelopsis grossedentata flavonoids and Tween 80 on feeding Nubian goats

**DOI:** 10.3389/fvets.2024.1411071

**Published:** 2024-07-12

**Authors:** Junhong Zhu, Ying Lu, Zhendong Gao, Yuqing Chong, Mengfei Li, Weidong Deng, Dongmei Xi

**Affiliations:** Yunnan Provincial Key Laboratory of Animal Nutrition and Feed, Faculty of Animal Science and Technology, Yunnan Agricultural University, Kunming, China

**Keywords:** Ampelopsis grossedentata flavonoids, Tween 80, Nubian goats, growth performance, blood indexes, rumen microbiota

## Abstract

**Introduction:**

The ban on antibiotics in animal husbandry underscores the crucial need for safe, natural feed additives. This study investigates the effects of Ampelopsis grossedentata flavonoids (AGF) and Tween 80 on the growth performance, blood indexes, and rumen microbiota of Nubian goats, evaluating their potential as alternative feed additives in livestock management.

**Methods:**

Thirty-two goats were randomly divided into four groups. The control group (CON group) was provided with a basal diet, while the experimental groups received diets supplemented with various dietary additives for a duration of 100 days: either a basal diet supplemented with 25 mg/kg of monensin (MN group), a basal diet containing 2.0 g/kg of Ampelopsis grossedentata flavonoids (AGF group), or a basal diet containing 7.5 mL/kg of Tween 80 (TW group). Blood and rumen fluid samples were collected for analysis at the end of the feeding period. Growth performance was monitored through regular weighing and feed intake measurements. Blood indexes were analyzed using standard biochemical techniques, while the microbial composition of the rumen fluid was determined through high throughput 16S rRNA gene sequencing to assess microbial diversity and function. The effects of the dietary treatments on growth performance, blood indexes, and rumen microbial composition were then evaluated.

**Results:**

The AGF group exhibited significantly increased average daily gain, and decreased feed-to-gain ratio (*p* < 0.05). Blood indexes analysis revealed no differences between the CON and AGF groups, with both showing higher concentrations of triglyceride, low-density lipoprotein cholesterol, glutamic-pyruvic transaminase, alkaline phosphatase, and lactate dehydrogenase compared to the monensin group (*p* < 0.05). The TW group had significantly higher glucose, glutamic-oxaloacetic transaminase, and glutamic-pyruvic transaminase levels than the MN group (*p* < 0.05). Microbial diversity analysis revealed that the TW group had significantly greater alpha-diversity than other groups, while beta-diversity analysis showed closer similarity between the rumen microbiota of the AGF and CON groups. LEfSe analysis identified *Proteobacteria, Deferribacteres, Ehryarchaeoia*, and *Elusimicrobia* as biomarkers distinguishing the rumen microbiota among the groups. In conclusion, AGF supplementation increased the relative abundance of beneficial bacteria in the rumen of Nubian goats, and thus enhanced the growth performance. TW supplementation significantly increased rumen microbial diversity and abundance, suggesting benefits for rumen health despite poor palatability. These findings highlight the potential of AGF as a new green additive with important implications for the efficiency and development of animal husbandry.

## 1 Introduction

Antibiotic additives have previously been widely used in animal husbandry (1). However, due to issues like drug residues, their use has been banned in many countries (2). This prohibition has posed significant challenges to animal husbandry as a whole, underscoring the critical need to explore natural, green, and safe feed additives. This study aimed to assess the effects of two potential alternatives—Ampelopsis grossedentata flavone (AGF) derived from natural plant extracts and Tween 80, widely used as an emulsifier—as feed additives on growth performance, blood indexes and rumen microbiota in Nubian goats.

AGF, an acronym for Ampelopsis grossedentata flavonoids, represents a group of natural plant extracts derived from the Ampelopsis grossedentata vine, known for their significant antioxidative properties. Its main component is dihydromyricetin (3), which has various physiological functions such as anti-inflammation, anti-oxidative stress (4), antibacterial (5), anti-apoptosis (6), immune regulation (7), and plays an important role in improving animal growth performance and enhancing animal immunity (8). Recent reports indicate that AGF not only shows strong antioxidant activity, but also has excellent liver protective properties that can be developed into products aimed at anti-diabetes, antioxidants and liver protection (9–11). The study found that rattan tea prevented metabolic disorders caused by a high-fat diet by improving glucose homeostasis in rats. Wang and colleagues' research found that the antioxidant qualities of AGF play a protective role in maintaining the integrity of the intestinal barrier within the porcine epithelial cell line (12). Zhu et al. found that incorporating AGF into the diet not only enhances the microbial ecosystem but also holds significant potential for advancing the growth of juvenile livestock, thereby contributing to economic gains (13). However, the specific role of AGF as feed additives in ruminants still requires further investigation.

The chemical name of Tween 80 is polyoxyethylene sorbide monooleate. It can increase the amount of enzyme release and improve enzyme activity, promote the interaction between enzyme and substrate (14). It is widely used as an emulsifier in food processing (15). Food-grade Tween 80 is generally considered safe as a feed additive (16). Experiments have shown that Tween 80 has a certain regulatory effect on rumen microorganisms, which can enhance the activity of cellulase and promote the growth of non-cellulolytic bacteria (17, 18). Martha's study found that Tween 80 and its derivative oleic acid promoted the growth of corynebacterium accolens (19). Research has demonstrated that the addition of the surfactant Tween-80 effectively boosts the natural ability of bacteria to biodegrade naphthalene (20). However, only a few studies have verified the effect of Tween 80 in ruminants, with particularly few reports on its application in meat sheep production.

Therefore, the aim of this study was to comprehensively investigate the effects of dietary supplementation with AGF and Tween 80 on growth performance, blood indexes, and rumen microbiota in Nubian goats, as well as to evaluate the feasibility of these two additives as alternatives to monensin. Through this study, we hope to provide a theoretical basis for the promotion and application of these natural and safe feed additives in the animal husbandry industry.

## 2 Materials and methods

The study was conducted at the farm in Zhaotong County, Yunnan Province, China. Nestled in the mid-mountainous terrain of Houlong Mountain at coordinates N27°31′13″, E103°54′51″, the farm stands proudly at an elevation of 1,614 m. This locale experiences a climatic climate characterized by high humidity levels ranging from 20% to 60% RH, and a penchant for precipitation. Throughout the summer months, spanning June to September, the daily temperatures peak at an average temperature of 26°C. However, a conspicuous diurnal-temperature delta. The research proposal and the relevant experimental procedures were approved by the institutional Animal Care and Use Committee of Yunnan Agricultural University (Case Number: 202200301).

### 2.1 Animals and experiment design

**Figure 1:**
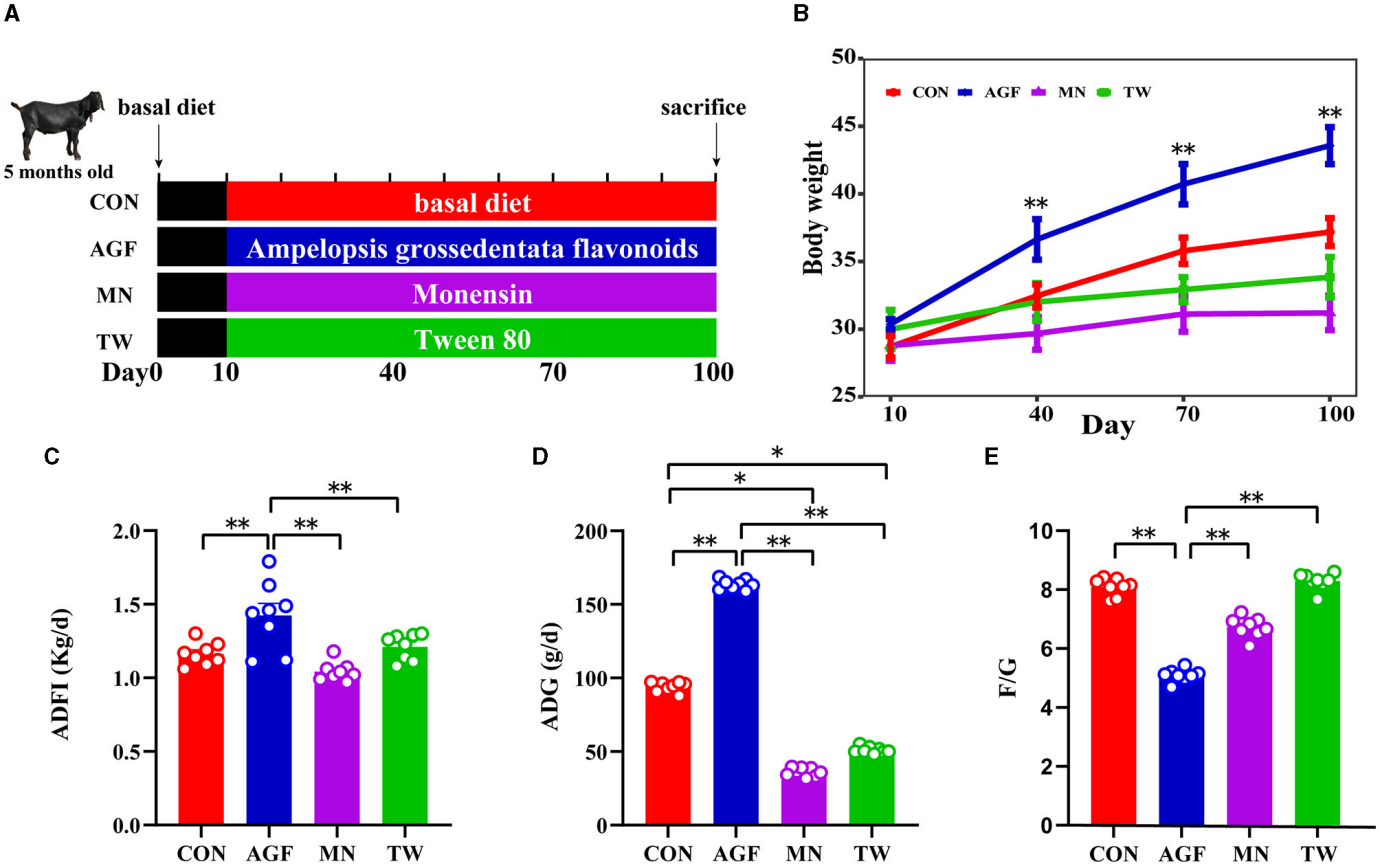
Influences of various additives on the growth performance in goats. **(A)** Schematic diagram of treatment of feed additives. **(B)** Changes in body weight of goats. **(C)** Average daily feed intake of goats. **(D)** The average daily gain of goats. **(E)** Feed to weight ratio of goats. * Indicates significant difference (*p* < 0.05), and ** indicates extremely significant difference (*p* < 0.01).

The study was carried out using a completely randomized design approach. Four groups were formed from a total of thirty-two Nubian goats (initial mean ± SE; 27 ± 1.39 kg body weight, and 5 ± 0.5 months of age) designated for fattening, with each group comprising eight goats (half male and half female). The basal diet formulations followed in this study were based on the guidelines provided by the NRC (21) and NY/T816-2004 (Table 1). The control group (CON group) was fed a standard basal diet. In contrast, the experimental groups received variations of this diet: one with an addition of 2.0 g/kg of Ampelopsis grossedentata flavonoids (AGF group), another incorporating 25 mg/kg of monensin (MN group), and the last one enhanced with 7.5 mL/kg of Tween 80 (TW group). We uniformly feed and manage all experimental goats. Spanning 100 days, the experiment was initiated with a 10-day preparatory phase dedicated to vaccination, deworming, management adjustments, and dietary transitions. The specific arrangement is shown in Figure 1A. The goats were fed twice daily, at 8:00 and 16:00 h with free access to fresh water.

**Table 1:** Composition and nutrient level of experimental basal diet (dry matter basis).

**Ingredients**	**Content (%)**	**Nutrients**	**Content**
Silage corn	56.50	Metabolic energy (MJ/Kg)	11.00
Broken maize	26.50	Digestible energy (MJ/Kg)	13.45
Hay powder	10.50	Crude protein	15.60
WDGS	5.00	NDF	39.72
CaHPO_4_	0.15	ADF	27.00
NaHCO_3_	0.15	Calcium	0.68
NaCl	0.20	Total phosphorus	0.26
Premix	1.00		
Total	100.00		

The premix provided the following per kg of diets VA: 15,000 IU, VD: 2200 IU, VE: 50 IU, Fe: 55 mg, Cu: 12.5 mg, Mn: 47 mg, Zn: 24 mg, Se: 0.5 mg, I: 0.5 mg, Co: 0.1 mg.

### 2.2 Sample collection

On the 101^st^ day, at 07:00, we collected blood samples from the jugular veins of eight goats in each group, using vacutainer tubes. These samples were immediately spun down at 3,000 rpm and a temperature of 4°C. Following centrifugation, we separated the plasma and stored it at −20°C for future analysis. Additionally, on the same day, before feeding, we gathered rumen fluid samples from six goats in each group, selected for their weights being closest to the group's average, using a vacuum pump. For microbial analysis, we employed high-throughput sequencing to identify unique microbial sequences in the 16S region (22).

### 2.3 Growth performance

Daily records were kept of feed consumption. At the scheduled time points (07:00) on the 11^th^, 41^st^, 71^st^, and 101^st^ days, goats undergo weight assessment after fasting. The feed conversion ratio was determined by applying the standard formula.

### 2.4 Plasma physiology and biochemistry

An automated biochemistry analyzer (Roche, Basel, Switzerland) was utilized to measure plasma physiology and biochemistry indices. These included triglyceride (TG), total protein (TP), glucose (GLU), UREA, total cholesterol (TC), high-density lipoprotein cholesterol (HDL-CH), low-density lipoprotein cholesterol (LDL-CH), globulin (GLOB), albumin (ALB), alanine aminotransferase (ALT), aspartate aminotransferase (AST), alkaline phosphatase (ALP), and lactate dehydrogenase (LDH).

### 2.5 16S rRNA gene sequencing

Microbial DNA was extracted using the HiPure Soil DNA Kits (Magen, Guangzhou, China) according to the manufacturer's protocols. The NanoDrop 2000 spectrophotometer (Thermo Fisher Scientific, Madison, WI, USA) was employed for DNA quantification, ensuring the procurement of high-quality genomic DNA in adequate amounts (23). The V3-V4 regions of the 16S rDNA genes were then amplified by PCR, using specific forward (5′-CCTACGGGNGGCWGCAG-3′) and reverse (5′-GGACTACHVGGGTATCTAAT-3′) primers (24). PCR was used to amplify the ribosomal RNA gene, with the protocol starting at 94°C for 2 min, followed by 30 cycles of 98°C for 10 s, 62°C for 30 s, and 68°C for 30 s, culminating in a final extension at 68°C for 5 min. The resulting amplicons were then extracted from 2% agarose gels and underwent purification via the AxyPrep DNA Gel Extraction Kit (Axygen Biosciences, Union City, CA), as per the guidelines provided by the manufacturer. Quantification was carried out using the ABI StepOnePlus Real-Time PCR System (Life Technologies, Foster City, CA). After purification, the amplicons underwent paired-end sequencing on the Illumina platform (PE250) following standard procedures.

FASTP (version 0.18.0) (25) was employed to filter raw data from the Illumina platform, resulting in clean reads for subsequent assembly analysis. The software FLASH (version 1.2.11) (26) was utilized to merge clean reads into tags based on a minimum overlap of 10bp and a maximum mismatch rate of 2%. Following the filtering criteria outlined in the literature (27), QIIME (version 1.9.1) (28) was employed to further filter low-quality tags, yielding a set of high-quality clean tags. Utilizing the UCHIME algorithm (29) with a reference database (version r20110519), chimera checking was performed on the clean tags. The resultant clean tags, post chimera filtering, were subjected to subsequent analysis. Using UPARSE software (version 7.1) (24), operational taxonomic units (OTUs) underwent reclustering at a 97% sequence similarity threshold (30). Chimeric sequences were identified and removed employing the Uchime algorithm (31, 32).

Principal Component Analysis (PCA) based on OTU abundance tables was conducted using the R language Vegan package (version 2.5.3) (33). The species abundance heatmap was generated using the R language heatmap package (version 1.0.12) (34). Pearson correlation analysis of species was computed using the psych package (version 1.8.4) (35). The species correlation network graph was constructed using the R language graph package (version 1.1.2) (36). Alpha-diversity indices, including Chao1 and Simpson, were calculated using QIIME version 1.9.1 (28). Diversity indices, including dilution curves and rank-abundance curves, were visualized through the ggplot2 package in R (version 2.2.1) (37). Non-metric Multidimensional Scaling (NMDS) analyses were conducted using the vegan package (version 2.5.3) in R, with resulting plots generated through the ggplot2 package (version 2.2.1) (38, 39). Intergroup Upset analysis, aimed at identifying unique and shared species, was performed using the UpSetR package in R (40). The LEfSe software (version 1.0) was utilized to identify biomarker features within each group (41). PICRUSt2 (version 1.0) was employed to infer KEGG pathway analysis of the OTUs (35). Between-group differences were evaluated through Welch's *t-*test and Wilcoxon rank-sum test for pairwise comparisons, alongside Tukey's Honestly Significant Difference (HSD) test and Kruskal-Wallis H test for multiple-group analyses, all conducted using the Vegan package (version 2.5.3) in R (33).

### 2.6 Data analysis

The impact of four diets on goats' growth performance, plasma metabolites, rumen pH, and rumen microbiota was analyzed using a one-way ANOVA in the SPSS 25.0 program (IBM Corp, Armonk, NY, USA), with results presented as means ± pooled SE (42). LSD and Duncan's multiple range tests facilitated multiple comparisons of means, categorizing differences as extremely significant (*p* < 0.01), significant (*p* < 0.05), or non-significant (*p* > 0.05) based on *p* values.

## 3 Results

### 3.1 Growth performance

The initial body weight comparison indicated no significant differences between the CON, AGF, MN, and TW groups (*p* > 0.05). However, a 90-day intake of Ampelopsis grossedentata flavonoids led to a significant increase in body weight in goats compared to the CON group (Figure 1B). The average daily feed intake (ADFI) and average daily gain (ADG) in the AGF group were significantly higher than those in the CON group, as well as those in the MN and TW groups (*p* < 0.01; Figures 1C, D). Additionally, the feed-to-gain ratio (F/G) in the AGF group was significantly lower than that in the CON group, and also significantly lower than that in the MN and TW groups (*p* < 0.01; Figure 1E).

### 3.2 Blood indexes

**Figure 2:**
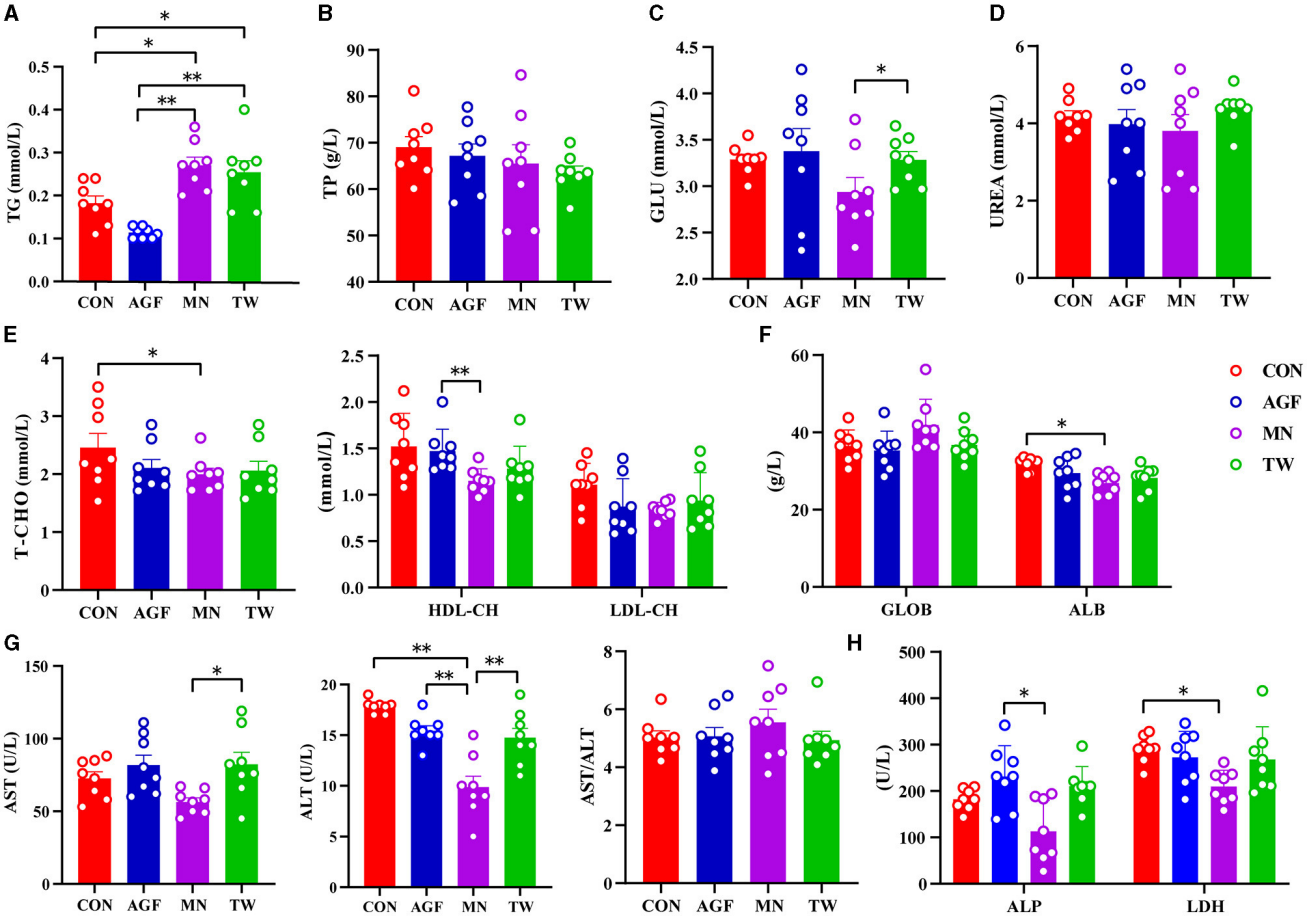
Effects of different additives on plasma metabolites of goats. **(A)** Hepatic triglyceride (TG) level. **(B)** Total protein (TP) level. **(C)** Glucose (GLU) level. **(D)** UREA level. **(E)** Cholesterol level. **(F)** Globulin (GLOB) and albumin (ALB) levels. **(G)** Alanine aminotransferase (ALT) and aspartate aminotransferase (AST) levels. **(H)** Alkaline phosphatase (ALP) and lactate dehydrogenase (LDH) levels. ^*^Indicates a significant difference (*p* < 0.05), ^**^Indicates an extremely significant difference (*p* < 0.01).

There were no significant differences in plasma TP and UREA concentrations between the four groups (*p*> 0.05) (Figures 2B, D). At the end of the experiment, there was no significant difference in plasma TG concentration between the AGF and CON groups (*p* > 0.05). However, the TG concentration in both groups was significantly higher than that in the MN and TW groups (*p* < 0.05; Figure 2A). There were no significant differences in plasma T-CHO and ALB concentrations between the AGF, TW, and CON groups (*p* > 0.05). However, the CON group had significantly higher T-CHO and ALB concentrations than the MN group (*p* < 0.05). The differences in plasma LDL-CH concentrations between the AGF, TW, and CON groups were not significant (*p* > 0.05). However, the LDL-CH concentration in the AGF group was significantly higher than that in the MN group (*p* < 0.05; Figures 2E, F). There were no significant differences (*p* > 0.05) in the plasma concentrations of GLU, AST, and ALT between the AGF, TW, and CON groups. The concentrations of GLU, AST, and ALT in the MN group were significantly lower than those in the TW group (*p* < 0.05), but there were no significant differences (*p* > 0.05) between the MN and AGF groups. In particular, the plasma concentration of ALT in the MN group was significantly lower than that in the CON and AGF groups (Figures 2C, G). The concentrations of ALP and LDH in MN group were significantly lower than those in AGF group and CON group, respectively (*p* < 0.05) (Figure 2H).

### 3.3 Composition and diversity of the rumen microbiota

#### 3.3.1 Rumen pH

Rumen pH was measured across different groups, yielding values of 6.71 ± 0.03 (CON), 6.62 ± 0.02 (AGF), 6.42 ± 0.05 (MN), and 6.61 ± 0.06 (TW), respectively. The analysis indicated that the various additives did not significantly affect rumen pH in goats (*p* > 0.05).

#### 3.3.2 Analysis of microbial diversity in the rumen

**Figure 3:**
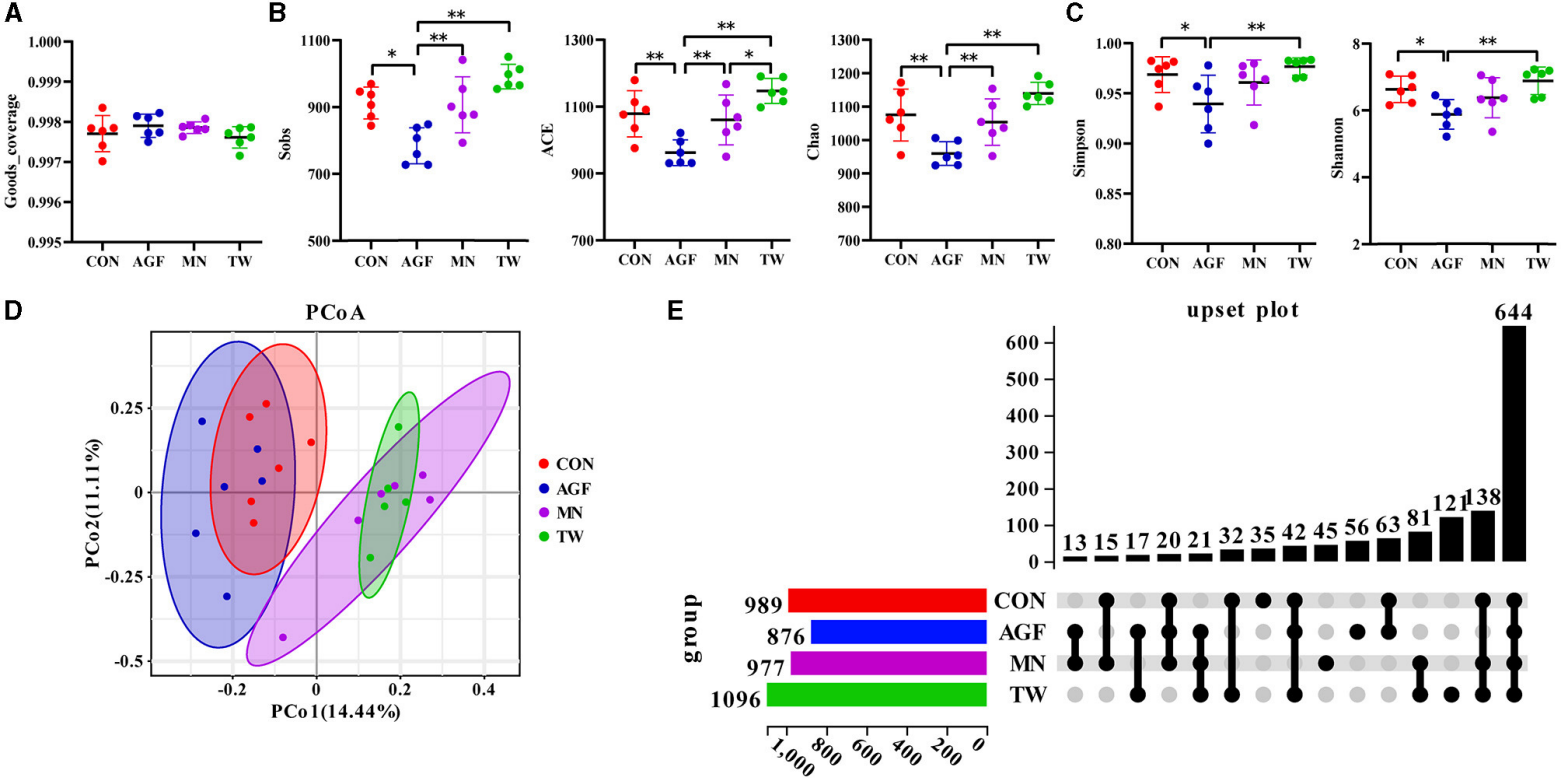
Diversity of rumen microbiota community as affected by additives. **(A)** Good's coverage index. **(B)** Sobs, ACE, and Chao1 indexes. **(C)** Shannon and Simpson indexes. **(D)** PCoA analysis. **(E)** Upset plot diagram at the OTU level of rumen microbiota in *four* groups. ^*^Indicates a significant difference (*p* < 0.05), ^**^Indicates an extremely significant difference (*p* < 0.01).

The Good's coverage of this sequencing exceeded 99%, ranging from 99.70% to 99.84% (Figure 3A). A diversity analysis was performed on OTUs to further understand the richness and diversity of microbial composition among the CON, AGF, MN, and TW groups. Compared to the CON group, all five α-diversity indices in the AGF group were significantly lower (*p* < 0.05). In contrast, there were no significant differences in the five α-diversity indices between the TW and CON groups (*p* > 0.05). The α-diversity in the AGF group was significantly lower than that in the MN and TW groups (*p* < 0.05). The microbial species richness in the AGF group was significantly decreased (*p* < 0.05; Figure 3B), whereas there was no significant difference in evenness (*p* > 0.05; Figure 3C). Principal Coordinate Analysis (PCoA) is a method of dimensionality reduction that employs a two-dimensional plane to visualize the distances between samples. Coordinates 1 represents 14.44% of the variation and coordinates 2 represents 11.11% of the variance (Figure 3D). The rumen microbial composition exhibited greater similarity between the TW and MN groups.

#### 3.3.3 OTU cluster

Following the exclusion of tags deemed of low quality or lacking biological significance, analysis of the 32 samples yielded a total of 2,879,868 effective tags. Each sample generated between 110,629 and 128,204 effective tags, with an effective rate of over 92% (Supplementary Table S1). A total of 989, 876, 977, and 1,096 operational taxonomic units (OTUs) were observed in the CON, AGF, MN, and TW groups, with 35, 56, 45, and 121 OTUs being unique to the CON, AGF, MN, and TW groups, respectively (Figure 3E). There were 644 OTUs common to all four groups, and intersections of CON and AGF, MN, TW groups contained 63, 15, and 32 OTUs, respectively (Figure 3E).

#### 3.3.4 Microbial composition

**Figure 4:**
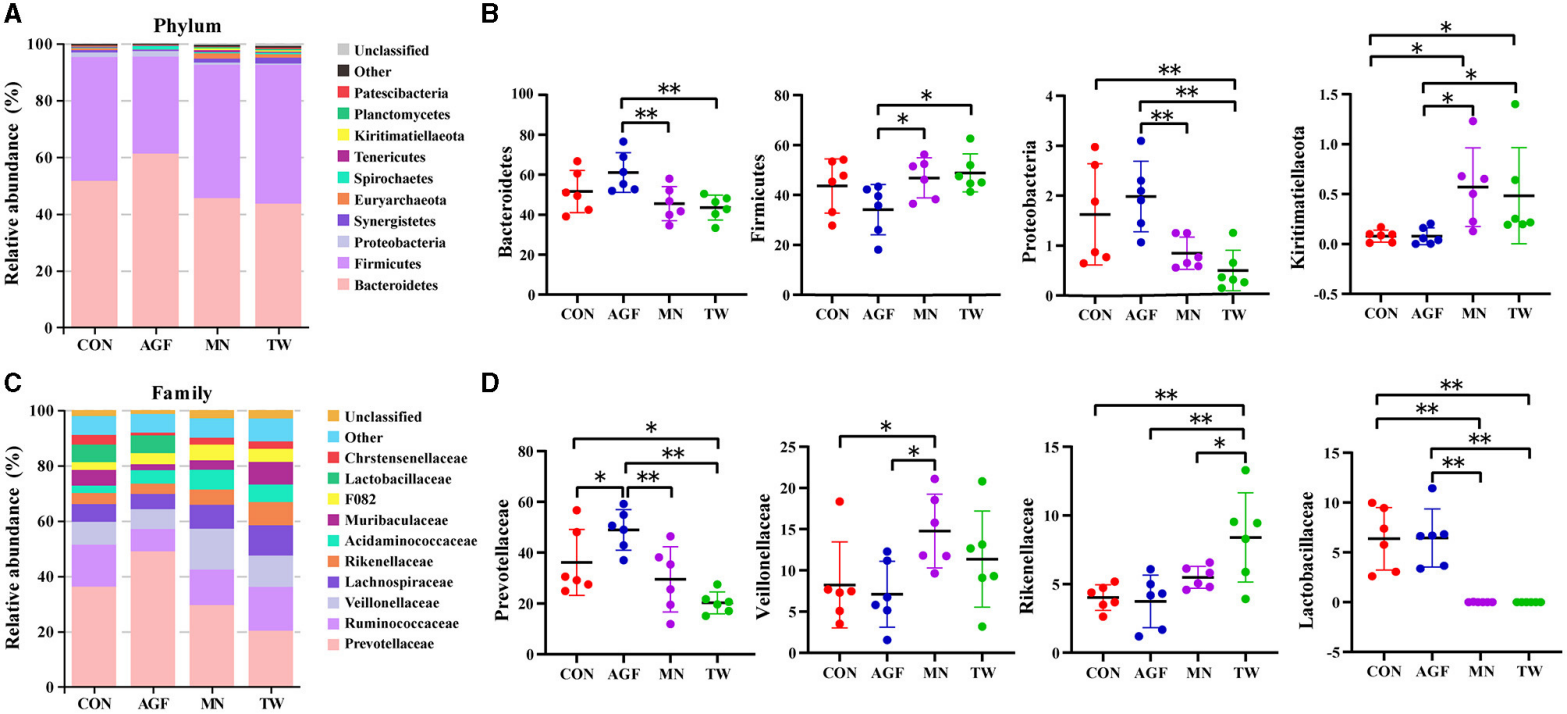
Species composition analysis of rumen microorganisms of goat at phylum and family level. **(A)** Top 10 dominant species at phylum level. **(B)** Dominant species of significant differences at phylum level. **(C)** Top 10 dominant species at family level. **(D)** Dominant species of significant differences at family level. ^*^Indicates a significant difference (*p* < 0.05), ^**^Indicates an extremely significant difference (*p* < 0.01).

Within the phylum level, *Bacteroidetes* and *Firmicutes* constituted the predominant phyla, collectively accounting for over 90% of the microorganisms across all four groups (Figure 4A), and their relative abundances ranging from 33.38% to 76.59% and 18.07% to 56.22%, respectively (Supplementary Table S2). Compared to the CON group, there were no significant differences in the relative abundance of *Bacteroides* and *Firmicutes* in the AGF and TW groups (*p* > 0.05). Additionally, the relative abundances of *Proteobacteria* and *Kiritimatiellaeota* in the AGF group did not show significant differences (*p* > 0.05). However, in the TW group, the relative abundance of *Proteobacteria* decreased significantly, while the relative abundance of *Kiritimatiellaeota* increased significantly (*p* < 0.05). Compared to the MN group, the relative abundances of *Bacteroides* and *Proteobacteria* were significantly higher in the AGF group (*p* < 0.01), whereas the relative abundances of *Firmicutes* and *Kiritimatiellaeota* were significantly lower (*p* < 0.05). There were no significant differences in the relative abundances of these taxa in the TW group (*p* > 0.05) (Figure 4B).

Within the family level, *Prevotellaceae* emerged as the dominant microbiota (Figure 4C). Compared with CON group, the relative abundance of *Prevotellaceae* in AGF group was significantly increased, while that in TW group was significantly decreased (*p* < 0.05). Compared with MN group, the relative abundance of *Prevotellaceae* in AGF group was significantly increased (*p* < 0.05), but not in TW group (*p* > 0.05). Compared with CON group, the relative abundance of *Veillonellaceae, Rikenellaceae*, and *Lactobacillaceae* in AGF group was not significantly different (*p* > 0.05), while the relative abundance of *Rikenellaceae* in TW group was significantly increased (*p* < 0.05). The relative abundance of *Lactobacillaceae* was significantly decreased (*p* < 0.01). Compared with the MN group, the relative abundance of *Veillonellaceae* in AGF group was significantly decreased, and the relative abundance of *Lactobacillaceae* was significantly increased (*p* < 0.05). The relative abundance of *Rikenellaceae* in TW group was significantly increased, and the relative abundance of *Lactobacillaceae* was significantly decreased (*p* < 0.05) (Figure 4D).

**Figure 5:**
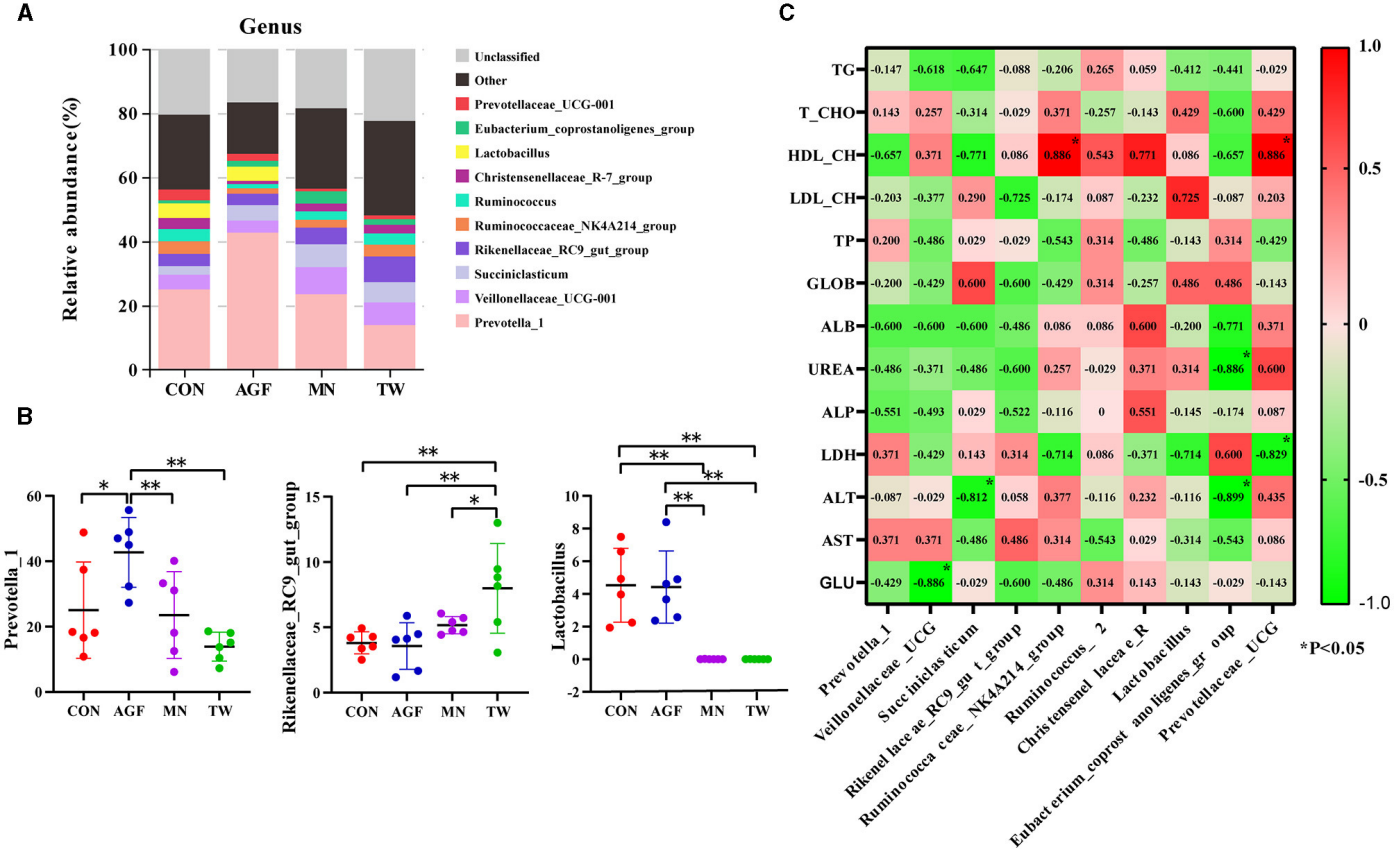
Species analysis at genus level. **(A)** Top 10 dominant species at genus level. **(B)** Dominant species of significant differences at genus level. **(C)** Analysis of the correlation between dominant genera in the rumen microbiota and plasma metabolites. ^*^Indicates a significant difference (*p* < 0.05), ^**^Indicates an extremely significant difference (*p* < 0.01).

Within the genus level, compared with CON group, the relative abundance of *Prevotella_1* in AGF group is significantly higher (*p* < 0.05), the relative abundance of *Rikenellaceae_RC9_gut_group* and *Lactobacillus* in TW group is not significantly different, and the relative abundance of *Prevotella_1* in TW group is not significantly different (*p* > 0.05) (Figures 5A, B). The relative abundance of *Rikenellaceae_RC9_gut_group* was significantly increased, and the relative abundance of *Lactobacillus* was significantly decreased (*p* < 0.05). Compared with MN group, the relative abundance of *Prevotella_1* and *Lactobacillus* in AGF group was significantly increased (*p* < 0.05), while the relative abundance of *Rikenellaceae_RC9_gut_group* was not significantly different (*p* > 0.05) (Figure 5B).

Correlation analysis was conducted between dominant rumen microbial genera in AGF group and various physiological and biochemical parameters in plasma, and the results were shown in Figure 5C. *Veillonellaceae_UCG-001* is significantly positively correlated with GLU (*p* < 0.05). *Succiniclasticum* is significantly negatively correlated with ALT (*p* < 0.05). The *Ruminococcaceae_NK4A214_group* and *Prevotellaceae_UCG-001* exhibited a significant positive correlation with HDL-CH (*p* < 0.01). Conversely, the *Eubacterium_coprostanoligenes_group* showed a significant negative correlation with UREA and AST (*p* < 0.01).

#### 3.3.5 Analysis of rumen differential microbiota

**Figure 6:**
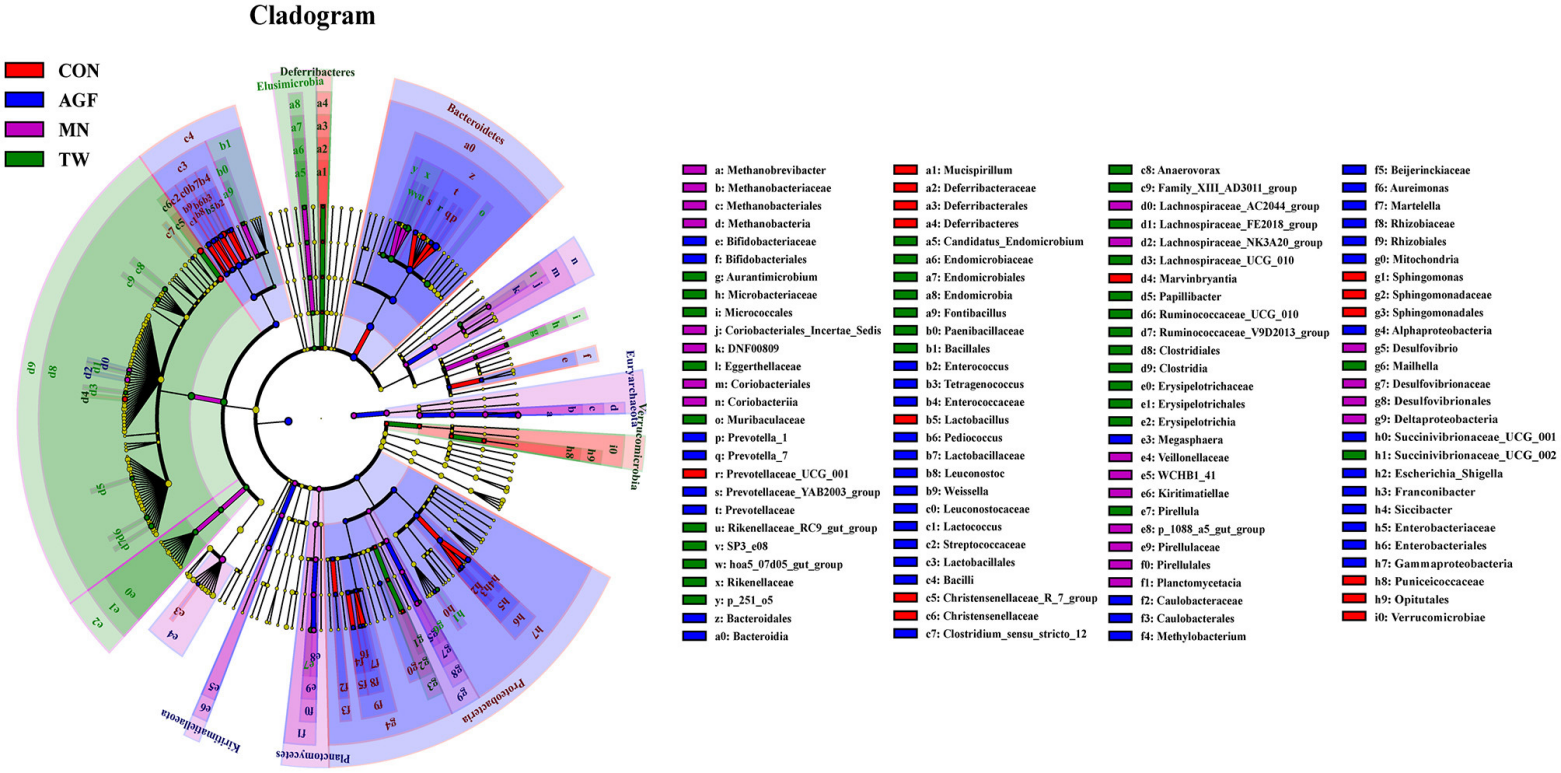
Analysis of indicator species using LEfSe (Linear discriminant analysis Effect Size).

In the AGF, CON, MN, and TW groups, a total of 117 biomarkers across various taxonomic levels were identified. Notably, *Proteobacteria, Deferribacteres, Euryarchaeota*, and *Elusimicrobia* emerged as key taxa that contributed to the observed differences in rumen microbiota among these groups (Figure 6). In the AGF and MN groups, a total of 112 biomarkers across various taxonomic levels were identified. Notably, *Prevotellaceae* and *Clostridia* emerged as key taxa that contributed to the observed differences in rumen microbiota among these groups (Supplementary Table S3). In the AGF and CON groups, a total of 24 biomarkers across various taxonomic levels were identified. Notably, *Clostridia* and *Pseudomonadales* emerged as key taxa contributed to the observes differences in rumen microbiota among these groups (Supplementary Table S4). In the TW and MN groups, a total of 12 biomarkers across various taxonomic levels were identified. Notably, *Erysipelotrichia* and *Bacterium_VCD2007* emerged as key taxa contributed to the observes differences in rumen microbiota among these groups (Supplementary Table S5). In the TW and CON groups, a total of 92 biomarkers across various taxonomic levels were identified. Notably, *Rikenellaceae* and *Prevotellaceae* emerged as key taxa contributed to the observes differences in rumen microbiota among these groups (Supplementary Table S6).

#### 3.3.6 Microbiota functional profile prediction

**Figure 7:**
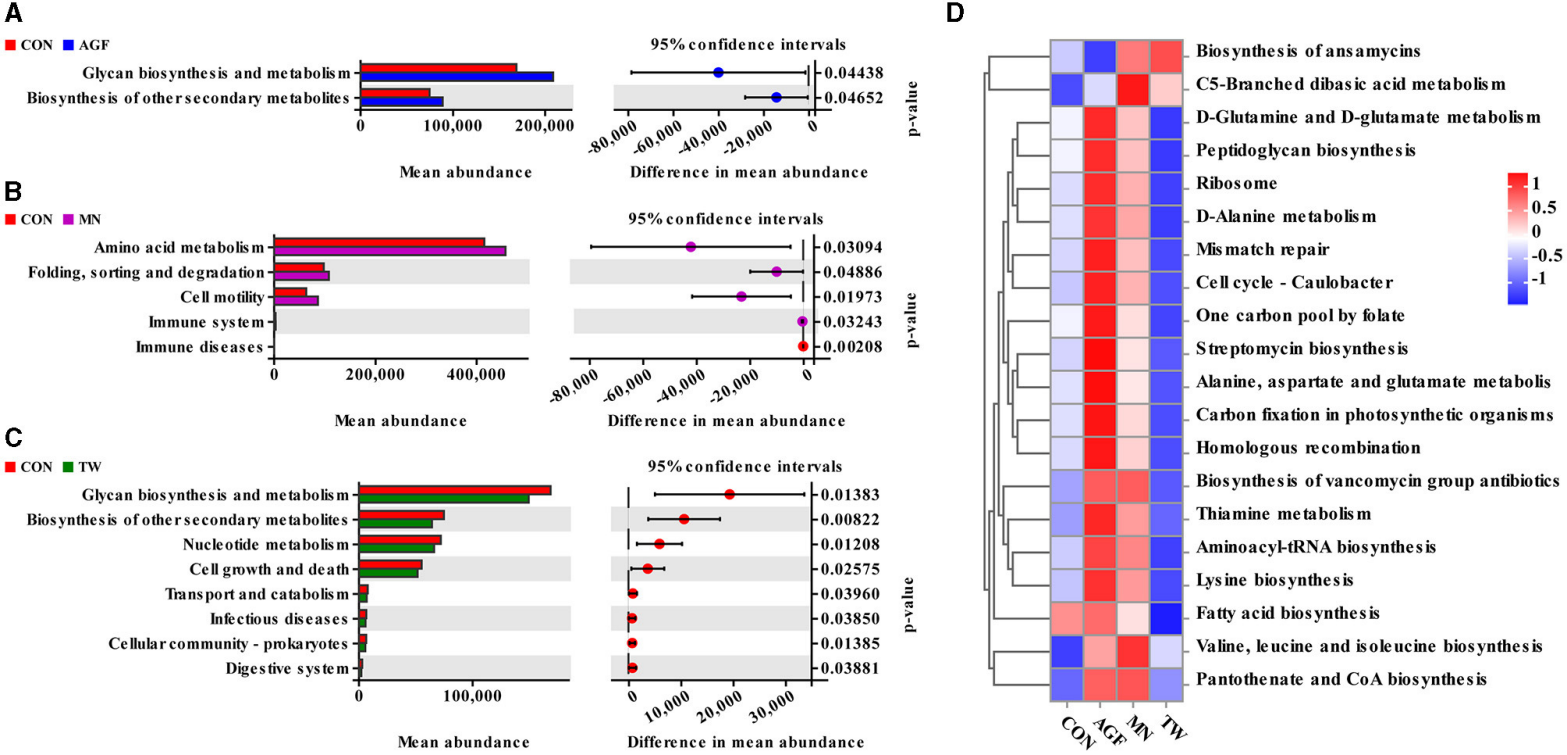
Forecasting the functions of microbiota using the PICRUSt2 platform. **(A)** Markedly distinct signaling pathways at level 2 between the AGF group and the CON group (*p* < 0.05). **(B)** Markedly distinct signaling pathways at level 2 between the MN group and the CON group (*p* < 0.05). **(C)** Markedly distinct signaling pathways at level 2 between the TW group and the CON group. **(D)** Level 3 heat map.

Functional prediction of the rumen microbiota was performed on the PICRUSt2 platform. Welch's *t*-test was applied to identify significant differences in functional pathways among the four groups at level 2. Two functional categories exhibited significant differences between the AGF and CON groups (*p* < 0.05). In the AGF group, both glycan biosynthesis and metabolism, and the biosynthesis of other secondary metabolites, were significantly elevated compared to the CON group (*p* < 0.05) (Figure 7A). Significant disparities were observed across five functional categories between the MN and CON groups (*p* < 0.05). In the MN group, the levels of amino acid metabolism, folding, sorting and degradation, and cell motility were significantly elevated compared to the CON group (*p* < 0.05) (Figure 7B). Eight functional classifications in the TW and CON groups showed statistically significant differences, with the TW group being significantly lower than the CON group (*p* < 0.05) (Figure 7C). At level 3, a total of twenty functional categories within the rumen microbiota of goats were predicted by the study. The AGF and MN groups showed similar enrichment in eighteen functional categories (Figure 7D).

## 4 Discussion

The comprehensive “ban” may have a certain impact on the production capacity of animal husbandry in the short term. At present, it has been found that plant extracts (43), probiotics (44), enzyme preparations (45), chitosan (46) and antimicrobial peptides (47) have the characteristics of high efficiency and no side effects. This research group has conducted experiments on natural plant additives and food additives, so as to overcome the defects of antibiotics to different degrees. Among these, Ampelopsis grossedentata flavonoids, a plant extract (48), and Tween 80, a non-ionic surfactant (49), both of which are non-toxic and harmless, are expected to become new pollution-free green feed additives. However, information on the use of AGF in ruminants remains scarce. Information regarding AGF application in ruminant animals is still limited. Thus, the objective of this study is to determine the effectiveness of AGF and TW by analyzing their influences on goats' growth performance, plasma metabolites, and rumen microbiota, all of which are key indicators of the animals' growth and health condition.

The improvement of goats' growth performance is closely related to ADFI and feed utilization. The results showed that both ADFI and ADG were higher in AGF group than in other groups, with the lowest F/G ratio, indicating high feed utilization and an effective fattening effect. This effectiveness is attributed to Ampelopsis grossedentata flavonoids' ability to alleviate oxidative stress responses, reduce gastrointestinal damage, and promote the gastrointestinal growth and development of goats (4). Contrary to expectations, the TW group demonstrated an adverse effect, differing from previous studies. For instance, McAllister et al. (16) observed that Tween 80 treatment in goats significantly reduced DMI and daily gain compared to untreated controls. Lee et al. (17) reported an increase in DMI and milk production in cows treated with Tween 80. The differing responses may be attributed to Ampelopsis grossedentata flavonoids' appealing fragrance enhancing feed intake, whereas Tween 80′s slightly unpleasant smell and bitter taste could deter goat from eating. Considering these factors, Ampelopsis grossedentata flavonoids show promise as a monensin substitute for enhancing growth performance, yet further rigorous testing is required.

Changes in plasma composition can reflect the nutritional level and organ function of ruminants (50). ALT and AST are two types of aminotransferases, play crucial roles in evaluating liver function, amino acids and proteins, metabolism, and indirectly influence the decomposition and synthesis of fats and sugars (51). Normally, AST levels in goats ranges from 40.0 to 123.0 U/L, but can increase when the liver is damaged (52). In this experiment, the plasma AST activity in all goat groups was within the normal range. ALP, an enzyme extensively found in various tissues such as the liver, bones, intestines, kidneys, and placenta, is secreted from the liver into the bile. It is associated with bone growth in animals, as well as the transport and utilization of sugars, lipids, proteins, and phosphoric acid. ALP levels reflect the growth and development of livestock and poultry (53). The ALP activity in goat plasma of the AGF group was significantly higher than in the MN group, warranting further investigation. HDL-CH, considered good cholesterol, transports lipids from tissues to the liver. In this experiment, HDL-CH levels in the AGF group were significantly higher than in the MN group. AGF is helpful to the absorption and transport of lipids in ruminants. In summary, AGF has shown to improve growth and development and lipid levels and lipid profiles in goats.

Rumen fluid pH reflects rumen fermentation status mainly by affecting rumen microbial activity. Studies have shown that dietary composition, saliva secretion, rumen fermentation product utilization, absorption efficiency, and before and after feeding can all cause rumen fluid pH changes, and dietary composition is the key factor causing pH changes (54). In addition, when rumen pH is 6.4 to 6.8, cellulose decomposing bacteria can achieve a suitable environment (55). When the pH of the rumen was 6.2 to 7.0, the rumen microbial ecosystem was relatively stable, which could ensure the normal fermentation of rumen (56). In this experiment, supplementing the diet with various feed additives did not markedly influence the pH of rumen fluid, which remained within the range of 6.4 to 6.8, indicating that the rumen environment was relatively stable, and the measured number of rumen microorganisms accurately reflected the microflora.

Rumen microorganisms and their hosts constitute a complex microecosystem, which plays a significant role in ruminant nutrition. In-depth study of rumen microecosystem is conducive to further understanding the mechanism of ruminant nutrition (57). Alpha-diversity refers to the diversity within a particular habitat or ecosystem. Good's Coverage reflects the sequencing saturation of the sample. Sobs, Chao1, and ACE indexes are mainly concerned with the species richness of samples. Simpson and Shannon comprehensively reflect the richness and evenness of species, so the level of the index will also be affected by evenness. The more evenly the species are distributed in the sample, the higher the diversity will be. In this experiment, the sequencing saturation of each group was sufficient. The Sobs, ACE, Chao1, Shannon, and Simpson indexes of the TW group were significantly higher than those of the other three groups. In this experiment, TW group had the most OTU and AGF group had fewer OTUs, which also corresponded to the diversity of results. In a nutshell, the findings suggest that the TW group exhibited the greatest species richness and evenness in the goat rumen fluid, thereby possessing the highest level of species diversity as well.

*Firmicutes* and *Bacteroidetes* emerged as the two predominant bacterial phyla in this study, corroborating the findings of earlier research (58). Among the top 10 phyla, the relative abundance of *Bacteroides* and *Proteobacteria* in the TW group is significantly lower than that in the AGF group, while the relative abundance of *Firmicutes* and *Kiritimatiellaeota* shows the opposite trend. *Bacteroides* play a key role in metabolizing polysaccharides and oligosaccharides, thereby furnishing nutrients and vitamins to both the host and other microbes residing in the intestines (59). Gut Firmicutes, stimulated by dietary fiber, and their metabolites display significant functions that promote health (60). *Prevotella_1* is the dominant genus, which is consistent with the results of previous studies, and its relative abundance in the AGF group is significantly higher than in other groups. Moreover, *Prevotella* is capable of utilizing starch, monosaccharides, and various non cellulosic polysaccharides (60). These results indicate that the synthesis and metabolism of sugars in the AGF group are stronger, which is consistent with the functional prediction in this experiment.

The *Rikenellaceae_RC9_gut_group*, part of the *Rikenellaceae* family, primarily ferments unabsorbed polysaccharides in the host's gut, leading to production of short-chain fatty acids like acetate, propionate, and butyrate (61). In this experiment, the relative abundance of *Rikenellaceae_RC9_gut_group* in the TW group was significantly higher than that in other groups. However, the glucose metabolism function of the TW group was significantly lower than that of the CON and AGF groups, which is speculated to be due to the lower abundance of *Rikenellaceae_RC9_gut_group* compared to *Prevotella_1* among the total bacterial genera. *Lactobacillus* is regarded as a beneficial bacterium, crucial for preserving the equilibrium of intestinal microbiota and thwarting the invasion of pathogens (62). The relative abundance of *Lactobacillus* in the AGF and CON groups was significantly higher than that in the TW and MN groups. In conclusion, the rumen microbial balance and glucose anabolic ability of the AGF group were relatively strong, and the diversity of AGF group was closer to that of the CON group, making it a good choice for green additives.

## 5 Conclusions

**Figure 8:**
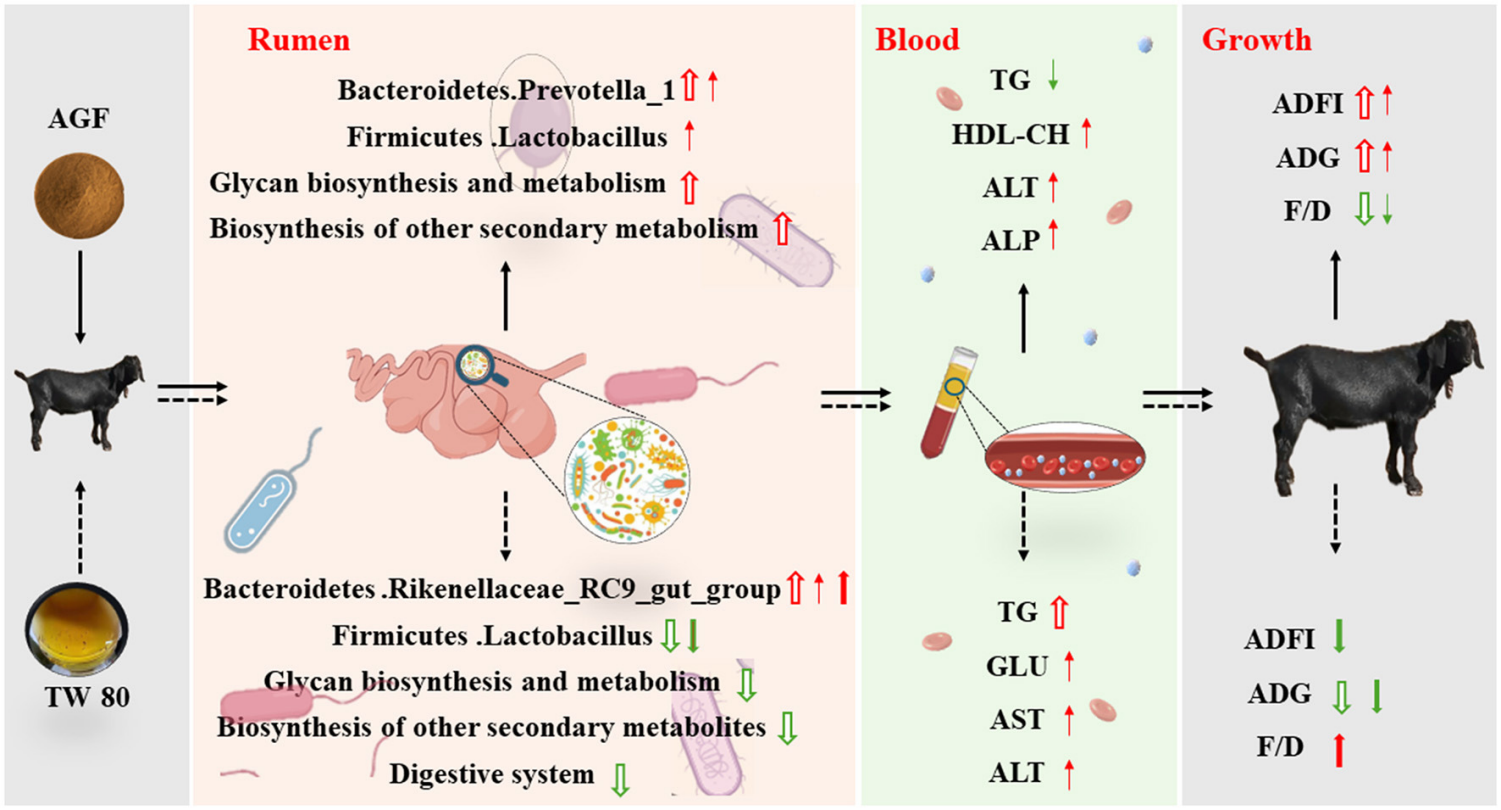
Regulation of Ampelopsis grossedentata flavonoids and Tween 80 on rumen microbial, plasma metabolites and growth performance of goats. Red arrows signify increases, while blue arrows indicate decreases. The hollow arrow indicates comparison with the CON group, the thin arrow indicates comparison with the MN group, and the thick arrow indicates comparison with the AGF group.

The adjustment diagram of this study is shown in Figure 8, which is drawn according to the experimental results. In conclusion, dietary addition of Ampelopsis grossedentata flavonoids can significantly increase the relative abundance of beneficial bacteria and affect the plasma physiological and biochemical indexes of goats. Further improve the average daily feed intake and daily gain of goats and reduce the ratio of feed to gain. Dietary supplementation of Tween 80 significantly increases the diversity and abundance of rumen microorganisms, thereby optimizing the microbial community and improving rumen health. However, despite these benefits, the abundance of beneficial bacteria is lower than in the control group, which suggests that improvements in rumen microbial diversity alone may not fully compensate for the lower palatability. Consequently, the feeding effect is poorer compared to the AGF group, indicating that the feeding method needs further improvement. All in all, Ampelopsis grossedentata flavonoids show potential as a new green additive to replace monensin and reduce stress in animal husbandry.

## Data availability statement

The data presented in the study are deposited in the NCBI repository, accession number PRJna956984.

## Ethics statement

The animal studies were approved by Animal Care and Use Committee of Yunnan Agricultural University. The studies were conducted in accordance with the local legislation and institutional requirements. Written informed consent was obtained from the owners for the participation of their animals in this study.

## Author contributions

JZ: Conceptualization, Data curation, Formal analysis, Investigation, Methodology, Validation, Visualization, Writing – original draft, Writing – review & editing. YL: Conceptualization, Investigation, Validation, Writing – original draft, Writing – review & editing. ZG: Investigation, Software, Writing – review & editing. YC: Investigation, Software, Writing – review & editing. ML: Formal analysis, Investigation, Writing – review & editing. WD: Investigation, Resources, Writing – review & editing. DX: Data curation, Funding acquisition, Methodology, Supervision, Writing – review & editing.
